# Protocol for a systematic review and meta-analysis of the efficacy of acupuncture and electroacupuncture against chemotherapy-induced peripheral neuropathy

**DOI:** 10.1097/MD.0000000000015098

**Published:** 2019-04-05

**Authors:** Man-Suk Hwang, Hye-Yoon Lee, Jin-Hyun Lee, Tae-Young Choi, Jung-Han Lee, Youn-Seok Ko, Sung-Youl Choi, Tae-Yong Park

**Affiliations:** a Third Division of Clinical Medicine, School of Korean Medicine, Pusan National University; b National Clinical Research Center for Korean Medicine, Pusan National University Korean Medicine Hospital, Yangsan; c Institute for Integrative Medicine, Catholic Kwandong University International St. Mary's Hospital, Incheon; d Medical Research Division, Korea Institute of Oriental Medicine, Daejeon; e Department of Rehabilitation Medicine of Korean Medicine, College of Korean Medicine, Wonkwang University, Iksan; f Department of Rehabilitation Medicine of Korean Medicine, College of Korean Medicine, Woo-Suk University, Jeonju, Jeonbuk; g Department of Neuropsychiatry, College of Korean Medicine, Gachon University, Seongnam, Gyeonggi, Republic of Korea.

**Keywords:** acupuncture, chemotherapy-induced peripheral neuropathy, CIPN, electroacupuncture, protocol, systematic review

## Abstract

**Introduction:**

Chemotherapy-induced peripheral neuropathy (CIPN) occurs in approximately 30% to 40% of patients who receive chemotherapy; however, standardized treatment for CIPN has not yet been developed. Acupuncture is widely used to treat CIPN in Korea, but its efficacy has not been investigated. The purpose of this study is to review the current literature on the efficacy of acupuncture and electroacupuncture (EA) in treating CIPN.

**Materials and Methods:**

We will perform a literature review using the relevant databases, including MEDLINE, Embase, the Allied and Complementary Medicine Databases (AMED), and China National Knowledge Infrastructure (CNKI), as well as Korean databases, including the National Digital Science Library (NDSL), Oriental Medicine Advanced Searching Integrated System (OASIS), DBpia, and Korean studies Information Service System (KISS). Randomized controlled trials describing treatment of CIPN symptoms with acupuncture or EA will be included. The primary outcomes will be scores on a visual analog scale and a numeric rating scale for neuropathic pain. We will also assess the risk of bias by evaluating the available studies using the tools of the Cochrane Collaboration and carry out a meta-analysis.

**Ethics and dissemination:**

Ethical approvals and patient consent are not necessary because the meta-analysis will be based on published research. We will submit our meta-analysis to a peer-reviewed journal for publication.

**PROSPERO registration number:**

CRD42018111509

## Abstract 2

Supplemental Digital Content is available in the text

## 1 Introduction

New cancer diagnoses are expected to increase in the future, from 14 million in 2012 to 22 million over the next 20 years.^[1]^ Chemotherapy is a type of cancer treatment where cytotoxic chemicals are administered, typically intravenously, to eradicate or reduce the tumor.^[2]^ Some of the major side effects of chemotherapy, including nausea, and vomiting,^[3]^ have received considerable attention, whereas peripheral neuropathy has received relatively limited attention.

Chemotherapy-induced peripheral neuropathy (CIPN) is one of the most common side effects in patients receiving chemotherapy.^[4]^ For example, side effects are observed in approximately 30% to 40% of patients who receive neurotoxin chemotherapy.^[5]^ Generally, it is more severe in the lower extremities than in the upper extremities and tends to appear bi-laterally.^[5]^ The main symptoms are neuropathic pain, numbness, a burning sensation and tingling in the skin, and weakness in muscles. A clear pathology of CIPN has not yet been established, and therefore, there are no standardized prevention and treatment agents that have been approved by the US FDA. In cases of CIPN where chemotherapy is in progress, it may be necessary to reduce the amount of anti-cancer drugs or, in severe cases, to temporarily suspend treatment.^[6]^ This not only reduces the effectiveness of chemotherapy but also increases the duration of cancer treatment. Central nervous system drugs, such as pregabalin and gabapentin, can be used for temporary symptom relief;^[7]^ however, these treatments have low symptom-reducing effects, can cause side effects such as dizziness and drowsiness,^[8]^ and cannot be administered at high doses due to narrow therapeutic windows.^[9]^

Acupuncture, a major form of traditional Korean medicine (TKM), has been used as one of adjunctive therapies to alleviate the symptoms of CIPN. Previous studies have reported that acupuncture is clinically effective against CIPN^10,11^, improves nerve conduction velocity^12^, and has persistent effects for 1 month.^[11]^ Studies on electroacupuncture (EA), which refers to electric stimulation applied on the acupuncture needle, were introduced relatively late, but are their use continues to increase.^[13,14]^ However, there is controversy about the effect of EA, because conflicting research results have been reported. EA was reported to be effective in relieving CIPN and improving quality of life and had no influence on immune function;^[15]^ while another study reported that EA was not more effective than the placebo.^[16]^

On that basis, our research team is planning a practical clinical study investigating the validity of CIPN treatment through TKM techniques, including acupuncture and EA. Before this study, however, a systematic review of the use of acupuncture and EA to treat CIPN is needed, this will inform the planned study as well as future practical clinical studies.

## 2 Methods and analysis

### 2.1 Study inclusion criteria

All studies involving randomized controlled trials investigating the use of acupuncture to manage CIPN will be eligible for inclusion in the review, without any restriction on publication status. All patients who have been diagnosed with CIPN will be included, regardless of their age, sex, race, or background. The experimental interventions will include acupuncture used for the treatment of CIPN, including manual acupuncture and EA. Studies that compare acupuncture used in conjunction with another active therapy to the other therapy alone will also be included. The control interventions will be conventional therapy, including physiotherapy and medication; TKM or alternative therapy other than acupuncture, including herbal medicine; or no treatment. The results of studies that used visual analog scales and numeric rating scales for the neuropathic pain of CIPN will be assessed to provide summary statistics, which will be the primary outcome of our study. The secondary outcomes include

(1) efficacy rate (number of patients who improved);
(2) nerve conduction velocity; and
(3) adverse events.

### 2.2 Search methods for the identification of studies

The following databases will be searched from their inception to September 2018: MEDLINE, Embase, the Allied and Complementary Medicine Databases (AMED), and China National Knowledge Infrastructure (CNKI), as well as Korean databases, namely the National Digital Science Library (NDSL), Oriental Medicine Advanced Searching Integrated System (OASIS), DBpia, and Korean studies Information Service System (KISS). The detailed search strategy used in the PubMed database will be presented in Appendix A. The equivalent search terms will be translated into the appropriate language for the database. We will search the reference lists of previously published reviews for potential qualifying studies. We will also manually search the proceedings and published papers from relevant conferences.

### 2.3 Data collection and analysis

#### 2.3.1 Selection of studies

**Figure 1:**
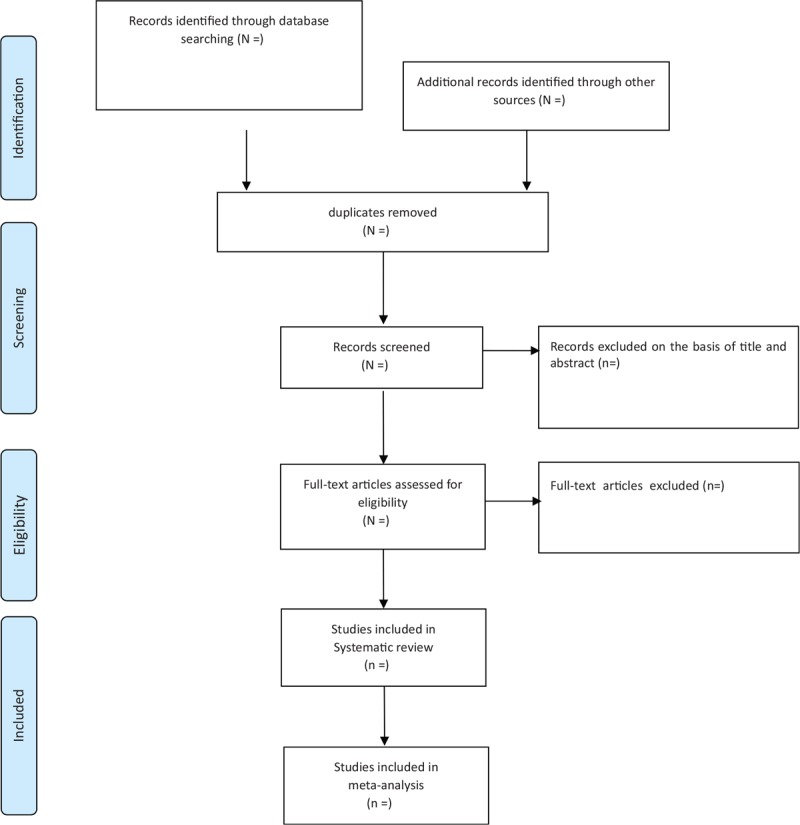
PRISMA diagram for the included studies. CR = conventional rehabilitation, IR = integrated rehabilitation.

Researchers will import the retrieved literature into Endnote X8 to eliminate redundancies. Articles that are noticeably inapplicable based on reading the titles and abstracts will be deleted. The final list of articles will be converted to Microsoft Excel format. Two researchers will independently conduct their own literature searches and will check whether each study is suitable for our analysis. Detailed reasons for eliminating studies will be documented. Disagreements will be resolved through discussion with the 2 reviewers. If an agreement cannot be reached, an independent reviewer will be consulted. Details of the study selection procedure are displayed in a PRISMA flowchart (Fig. 1).

### 2.4 Data extraction and management

Two researchers will review the text of each study and extract data on research design, blinding, applied intervention, controls, outcome indicators, major results, and other detail information, which will be analyzed and tabulated. If necessary, we will contact authors to get more information.

### 2.5 Assessment of risk of bias in included studies

The risk of bias will be assessed independently by 2 authors based on the Cochrane “Risk of Bias” assessment, which consists of random sequence generation, allocation concealment, blinding of participants and outcome assessors, incomplete outcome data, and other sources of bias. The risk of bias will be classified as high, unclear, or low.^[17]^

### 2.6 Measures of treatment effect

To summarize the effects of acupuncture treatment for each study, relative risk will be used when the result is dichotomous data. In cases with continuous data, the mean differences or the standard mean differences will be used. The effect sizes will be displayed using 95% confidence intervals.

### 2.7 Dealing with missing data

If data are missing or insufficient, we will contact the author by e-mail or telephone to obtain the necessary information. If we fail to recover sufficient data, the data will be discarded. We will conduct our analysis based on available data, and the potential impact of missing data will be discussed.

### 2.8 Assessment of heterogeneity

The heterogeneity of the study results will be analyzed using a χ^2^ test and determined using the I^2^ value. If the I^2^ is less than 50%, the statistical heterogeneity between tests can be ignored, and the effect size will be estimated using a fixed-effect model. If I^2^ exceeds 50%, there is considerable heterogeneity between tests, so a subgroup analysis will be performed to identify possible reasons.

### 2.9 Assessment of reporting bias

If more than 10 studies are included, publication bias will be examined by drawing a funnel.^[18,19]^ Studies with a small number of subjects often have different effect sizes because they are less accurate, while studies with many subjects typically show a small range of effect sizes because of their high accuracy. If there is no publishing bias, the funnel is symmetrical. A regression test developed by Egger et al^[19]^ will be used as a complementary method to represent the symmetry of the funnel shape using a *P* value.

#### 2.9.1 Data synthesis

RevMan ver 5.3 (Cochrane) software will be used to perform the meta-analysis. If there is no statistical heterogeneity between the results, a fixed-effect model will be used for the meta-analysis. If there is statistical heterogeneity, further analysis of the sources of heterogeneity will be conducted. After the obvious effect of clinical heterogeneity is ruled out, a random effects model will be used for the meta-analysis.

#### 2.9.2 Subgroup analysis

If there is considerable heterogeneity in the clinical research, we will conduct a subgroup analysis based on the types of acupuncture treatment and the clinical characteristics of the controls.

#### 2.9.3 Sensitivity analysis

Sensitivity analysis will be performed to determine the robustness of the review results. The main criteria will include the impact of sample size, methodological quality, and the effect of missing data.^[20]^

### 2.10 Grading the quality of evidence

Grading of Recommendations Assessment, Development, and Evaluation (GRADE) will be used to assess the quality of evidence for key outcomes. The quality of evidence will be classified into 4 levels: high, moderate, low, and very low.

## 3 Ethics and dissemination

Ethical approvals and patient consent are not necessary because the meta-analysis will be based on published research. We will submit our meta-analysis to a peer-reviewed journal for publication.

## 4 Discussion

A previous systematic review revealed that 1 randomized controlled trial showed the effect of acupuncture on CIPN, but the quality of other included studies was relatively low so a confirmative conclusion could not be derived.^[21]^ In addition, this systematic review indicated that till date, only 1 animal study has reported on the neuropathic-pain relief effect of EA, suggesting that further clinical studies are required. The present study is expected to include more studies of higher quality that encompass the effect of EA as well as acupuncture.

This study will be limited to papers written in English, Chinese, and Korean due to language barriers. However, the results of this review will provide more evidence and will help clinicians make the best choice for patients.

## Author contributions

MSH and TYP designed the study. SYC, TYC, YSK, Jin-Hyun Lee and Jung-Han Lee developed the search strategy. MSH and HYL wrote the manuscript. All authors provided critical revisions of the protocol and approved the final manuscript.

**Methodology:** Jin-Hyun Lee, Tae-Young Choi, Jung-Han Lee, Youn-Seok Ko.

**Project administration:** Sung-Youl Choi, Tae-Yong Park.

**Supervision:** Sung-Youl Choi, Tae-Yong Park.

**Writing – original draft:** Man-Suk Hwang.

**Writing – review & editing:** Hye-Yoon Lee.

Hye-Yoon Lee orcid: 0000-0002-9486-1703.

## Supplementary Material

Supplemental Digital Content
