# Supplementary material for: Protocol for a systematic review and meta-analysis of the efficacy of acupuncture and electroacupuncture against chemotherapy-induced peripheral neuropathy
Source: Medicine (Baltimore). 2019 Apr 5;98(14):e15098. doi: 10.1097/MD.0000000000015098 (PMC6456145; doi:10.1097/MD.0000000000015098)
Supplement: Supplemental Digital Content [file medi-98-e15098-s001.pdf]

Search strategy

○ Medline

#1. Antineoplastic Agents [MeSH Terms]

#2. (chemotherapy or (antineoplastic agents) or (chemotherapeutic Anticancer drug) or (anticancer agent) or cisplatin or carboplatin or oxaliplatin or bortezomib or docetaxel or paclitaxel or taxane or taxotere or (organoplatinum compounds) or platinum or vincristine or (vinca alkaloids) or thalidomide or (proteasome inhibitor)) [Title/Abstract]

#3. #1 OR #2

#4. Peripheral Nervous System Disease [MeSH Terms]

#5. (neuralgia or paresthesia or hyperalgesia or (chemotherapy induced peripheral neuropathy) or CIPN or (peripheral neuropathy) or Polyneuropath or (chemotherapy induced neurotoxicity)) [Title/Abstract]

#6. #4 OR #5

#7. Acupuncture [MeSH Terms]

#8. Acupuncture therapy [MeSH Terms]

#9. Electroacupuncture [MeSH Terms]

#10. Acupoint [MeSH Terms]

#11. “Acupuncture and moxibustion” [Title/Abstract]

#12. OR/ #7- #11

#13. #3 AND #6 AND #12

○ EMBASE, AMED

- same to Medline
